# The effect of ovarian puncture on the endocrine profile of PCOS patients who undergo IVM

**DOI:** 10.1186/1477-7827-12-18

**Published:** 2014-02-24

**Authors:** Carolina Ortega-Hrepich, Nikolaos P Polyzos, Ellen Anckaert, Luis Guzman, Herman Tournaye, Johan Smitz, Michel De Vos

**Affiliations:** 1 Centre for Reproductive Medicine, UZ Brussel, Laarbeeklaan 101, 1090 Brussels, Belgium; 2 Laboratory of Clinical Chemistry and Radioimmunology, UZ Brussel, Laarbeeklaan 101, 1090 Brussels, Belgium

**Keywords:** Anti-Müllerian hormone, In-vitro maturation, Immature oocyte retrieval, Sex hormone-binding globulin, Total testosterone

## Abstract

**Background:**

To examine whether ovarian puncture for immature oocyte retrieval and in-vitro maturation (IVM) has an effect on the endocrine profile of patients with polycystic ovary syndrome (PCOS).

**Methods:**

Twenty-two consecutive patients with PCOS undergoing IVM treatment were included. Serum anti-Müllerian hormone (AMH), sex hormone-binding globulin (SHBG), total testosterone (TT) and luteinized hormone (LH) levels were analyzed at the start of the cycle, on the day of immature oocyte retrieval (OR) and at fixed intervals thereafter, for up to three months after OR.

**Results:**

Five days after OR circulating AMH, TT, calculated free testosterone (FTc), and LH levels were significantly reduced and circulating SHBG was significantly increased. Two weeks after OR, TT, FTc and LH remained reduced, whereas circulating AMH and SHBG levels recovered to pre-puncture values. Three months after OR, all circulating hormone levels had recovered to baseline values.

**Conclusion:**

Ovarian puncture for the retrieval of immature oocytes and IVM in patients with PCOS has a significant impact on the ovarian endocrine profile, but this impact is brief and transient.

## Background

In-vitro maturation (IVM) of oocytes is a mild-approach assisted reproductive technology (ART) whereby immature oocytes are retrieved from small antral follicles. Although life birth rates after IVM are still lower than after conventional hormone-driven methods of ART that encompass oocyte maturation in vivo, IVM has promising potential in patients who suffer from significant hormonal side effects and an increased risk of ovarian hyperstimulation syndrome (OHSS) if they undergo conventional ART. Different promising strategies have been used to reduce the risk of OHSS in patients with polycystic ovary syndrome (PCOS) including ovarian stimulation using a GnRH-antagonist protocol followed by a GnRH-agonist ovulation trigger [1], low-dose stimulation protocol using highly purified follicle-stimulation hormone [2] and treatment segmentation with elective freezing of all oocytes or embryos [3]. Although these strategies can reduce OHSS incidence significantly, they do not completely eliminate the occurrence of OHSS in high-risk patients [4]; in these patients, IVM is a potential viable method to prevent OHSS [5]. Therefore, IVM could be highly suitable for patients with polycystic ovary syndrome.

During an IVM cycle, egg collection is typically performed using multiple transvaginal punctures of unstimulated or minimally stimulated ovaries; a recent retrospective study suggested that this egg collection procedure may exhibit a similar effect to that of laparoscopic ovarian drilling (LOD) [6]. According to this trial, oocyte retrieval for IVM was associated with an increased number of mature oocytes and embryos in subsequent in vitro fertilisation (IVF) cycles, implying that these punctures may have improved the endocrinological profile of patients with polycystic ovaries. Laparoscopic ovarian drilling (LOD) is widely used to induce ovulation in patients with PCOS who fail to ovulate or conceive with clomiphene citrate (CC), although there is no evidence for a significant difference in rates of clinical pregnancy, live birth, or miscarriage in women with CC resistant PCOS undergoing LOD as compared to other medical treatments [7]. Nonetheless, in spite of the hypothesis that ovarian puncture of polycystic ovaries may have a drilling-like effect, no prospective study has evaluated the effect of ovarian puncture for immature oocyte retrieval and IVM on the endocrine profile in patients with PCOS. Therefore, we performed a prospective cohort study to examine the alteration of serum anti-Müllerian hormone (AMH) and other hormone levels in relation to the oocyte retrieval procedure in a consecutive series of patients with PCOS who underwent IVM treatment.

## Methods

### Patients

From January 2012 to July 2012, serial serum levels of endocrine parameters from 22 consecutive patients with PCOS undergoing non-hCG-triggered oocyte retrieval for IVM (IVM-OR) were prospectively analyzed. The inclusion criteria for study were as follows: women younger than 35 years old, diagnosed with PCOS undergoing IVM cycle. Patients were classified as having PCOS if they fulfilled the Rotterdam criteria for PCOS [8,9]. Patients with congenital adrenal hyperplasia, Cushing’s syndrome and androgenic-secreting tumors were excluded. Male factor infertility was not an exclusion criterion. Written informed consent was obtained from all patients before inclusion in the trial. The study was approved by local Ethical Committee.

### IVM cycle characteristics

In oligomenorrhoeic and amenorrhoeic patients, a withdrawal bleeding was induced using oral dydrogesterone (Duphaston®, Abott Products GmbH, Hannover, Germany; 10 mg daily for 5 days). All patients received transdermal 17ß-estradiol gel (Oestrogel®, Besins Healthcare, Paris, France) for endometrial preparation, at a starting dose of 4 mg daily for seven consecutive days, after which the dose was increased to 7.5 mg daily. After a mean interval of 10.7 ± 2.6 days of 17ß-estradiol gel administration all patients received 150 IU highly purified human menopausal gonadotropin (HP-hMG) (Menopur®; Ferring Pharmaceuticals A/S, Copenhagen, Denmark) daily for three consecutive days. Transvaginal oocyte retrieval was scheduled 42 hours after the last injection of HP-hMG and was performed using a single lumen 17 GA needle (Cook Medical, K-OPS-1230-VUB, Limerick, Ireland) at an aspiration pressure of 70 mm Hg. To avoid the retrieval of oocytes at divergent stages of maturation at the time of retrieval, no human chorionic gonadotropin (hCG) trigger was administered. Cumulus-oocyte complexes (COCs) were matured for 40 hours in MediCult IVM® System (Origio, Måløv, Denmark). Intracytoplasmic sperm injection (ICSI) and embryo transfer were performed as previously described [10,11].

### Measurements and follow-up

Serial blood sampling for the analysis of serum levels of AMH, total testosterone (TT), calculated free testosterone (FTc), sex hormone-binding globulin (SHBG), follicle stimulating hormone (FSH) and luteinized hormone (LH) was performed on day two of the IVM treatment cycle, on the day of IVM-OR (before the oocyte collection), five days after IVM-OR and two weeks after IVM-OR.

To extend the interval of follow-up after the IVM cycle, patients were invited to return to the clinic for further blood sampling three months after oocyte retrieval and a subanalysis was performed in those who did return.

### Assays

Serum AMH levels were measured with the Beckman Coulter Gen II ELISA kit (Beckman Coulter, Marseille, France). To circumvent inter-assay variability, all samples were processed simultaneously. Serum FSH, LH, estradiol (E2), progesterone, SHBG and total testosterone levels were measured on a Cobas 6000 immunoanalyser (Roche Diagnostics, Mannheim, Germany). Free testosterone was calculated from total serum testosterone and SHBG as measured by immunoassay and assuming a fixed albumin concentration as described previously [12].

### Statistical analysis

Data were analyzed using SPSS 20.0 statistical software. Normality of the distribution for each of the outcomes was examined using the Shapiro-Wilk test. Due to the lack of normality in the distribution, paired analysis was performed using the Wilcoxon Signed-Rank Test. Differences were considered statistically significant when P ≤ 0.05.

## Results

Table 1 shows baseline patient and hormonal characteristics of the study population.

**Table 1:** Baseline patient characteristics

	**Mean ± SD**
*Age (y)*	29.7 ± 3.5
*BMI (kg/m*^*2*^*)*	30.3 ± 9.0
*AFC*	20.2 ± 11.0
*FSH (IU/L)*	5.5 ± 1.2
*E2 (ng/L)*	47.3 ± 13.1
*LH (IU/L)*	7.9 ± 4.4
*Progesterone (μg/L)*	0.6 ± 0.2

*BMI* body mass index, *AFC* antral follicle count, *FSH* follicle stimulating hormone, *E2* Estradiol, *LH* Luteinizing hormone.

### Circulating hormone levels during the IVM cycle

On day two of the cycle (cd2), the mean basal levels of serum AMH, E2, TT, FTc and SHBG were 8.9 ± 4.0 μg/L, 47.4 ± 13.1 ng/L, 0.36 ± 0.13 nmol/L, 5.0 ± 3.6 pmol/L and 74.6 ± 59.1 nmol/L, respectively. After endometrial priming with transdermal 17ß-estradiol and a cumulative dose of 450 IU HP-hMG, a second blood sample was obtained on the day of IVM-OR: serum AMH levels were significantly reduced to 7.5 ± 3.2 μg/L (p = 0.005) and serum E2 and TT levels were significantly increased to 1098 ± 1831 ng/L (p < 0.001), 0.48 ± 0.21 nmol/L (p = 0.002, resp). FTc, SHBG and LH levels all remained unchanged on the day of OR compared to basal cd2 values [5.3 ± 2.9 pmol/L (p = 0.570), 85.1 ± 48.3 nmol/L (p = 0.082) and 10.2 ± 7.8 IU/L, p = 0.277, resp.].

Five days after IVM-OR, circulating AMH levels significantly dropped to 6.3 ± 3.2 μg/L (p = 0.001) compared to values at IVM-OR and circulating levels of TT, FTc and LH levels were also significantly reduced [0.28 ± 0.12 nmol/L (p < 0.001), 2.9 ± 1.8 pmol/L (p < 0.001) and 5.8 ± 4.1 IU/L (p = 0.023), resp.]. Circulating SHBG was significantly increased to 99.4 ± 57.3 nmol/L (p = 0.017).

These significant changes of circulating AMH and SHBG levels were no longer observed two weeks after OR: circulating AMH recovered to 7.6 ± 3.6 μg/L (p = 0.910) and SHBG recovered to 81.6 ± 54.4 nmol/L (p = 0.875). However, TT, FTc and LH remained significantly reduced two weeks after IVM-OR, compared to levels at IVM-OR [0.22 ± 0.1 nmol/L (p = 0.001), 2.7 ± 1.5 pmol/L (p = 0.002) and 3.7 ± 3.0 IU/L (p = 0.008), resp.] (Table 2).

**Table 2:** Endocrine profile after oocyte retrieval (OR) for IVM in PCOS

	**Basal**	**OR day (All values compared to basal values)**	**5 days after OR (All values compared to OR values)**	**2 weeks after OR (All values compared to OR values)**
*AMH (μg/L)*	8.9 ± 4.0	7.5 ± 3.2*	6.3 ± 3.2*	7.6 ± 3.6^NS^
*TT (nmol/L)*	0.36 ± 0.13	0.48 ± 0.21*	0.28 ± 0.12**	0.22 ± 0.1*
*FTc (pmol/L)*	5.0 ± 3.6	5.3 ± 2.9^NS^	2.9 ± 1.8**	2.7 ± 1.5*
*SHBG (nmol/L)*	74.6 ± 59.1	85.1 ± 48.3^NS^	99.4 ± 57.3*	81.6 ± 54.4^NS^
*LH (IU/L)*	7.9 ± 4.4	10.2 ± 7.8^NS^	5.8 ± 4.1*	3.7 ± 3.0*

Values represent mean and SD.

*AMH* Antimüllerian hormone, *TT* total testosterone, *SHBG* sex hormone-binding globulin, *FTc* calculated free testosterone, *LH* Luteinizing hormone, *FSH* Follicle-stimulating hormone. *P < 0.05; **P < 0.001; *NS* not statistically different.

Because of the significant impact of IVM-OR on serum androgens and LH levels two weeks after IVM-OR, patients were invited to have further blood sampling for hormone analysis three months after IVM-OR.

### Circulating hormone levels three months after IVM-OR

Blood samples for hormone analysis were obtained from five patients at three months’ follow-up. The remaining 17 patients were unavailable for this subanalysis: 10 patients had undergone a second IVM-OR procedure in the meantime, 2 patients were receiving hormonal treatment, 3 patients had become pregnant after the IVM treatment cycle and 2 patients were lost to follow-up.

Three months after IVM-OR, no significant differences were found when comparing circulating levels of AMH, TT, FTc, SHBG and LH to basal levels ^(a)^ or OR levels ^(b)^. Total testosterone, FTc and LH levels had recovered to 0.37 ± 0.16 nmol/L (p = 0.225^(a)^, p = 0.5^(b)^), 4.0 ± 2.4 pmol/L (p = 0.893 ^(a)^, p = 0.893 ^(b)^) and 5.8 ± 4.7 IU/L (p = 0.345 ^(a)^, p = 0.686 ^(b)^), respectively (Table 2).

## Discussion

To our knowledge, this is the first prospective study to examine the effect of ovarian puncture for retrieval of immature oocytes on the endocrine profile in patients with PCOS undergoing IVM treatment. The results of our study suggest that the effect of oocyte retrieval of immature oocytes on the endocrine profile of these patients is significant but transient and that ovarian puncture for IVM does not offer long-term prospects of an alteration of the endocrine profile in patients with PCOS, as observed after LOD. Although the exact mechanism of action of LOD is unknown, it is likely that multiple ovarian punctures may destroy ovarian androgen-producing tissue and by doing so result in reduced peripheral conversion of androgen into oestrogen [13]. This is further documented by the observation that LOD can restore ovulation, results in good pregnancy rates [14,15] and significantly reduces AMH levels [16], LH/FSH ratio, serum concentration of LH, testosterone and free androgen index. The effect of LOD on these endocrine parameters often persists for many years after the procedure [17].

Although gonadotropin doses in IVM cycles are typically much lower than those administered in conventional ART (c-ART) cycles, clinical protocols for IVM often include mild stimulation using low-dose gonadotropins and/or oral or transdermal estrogens [18-20] to increase endometrial thickness. In our study, patients received 14.7 ± 2.5 days of transdermal oestradiol and 450 IU HP-hMG cumulatively for endometrial priming before oocyte retrieval, resulting in mean serum E2 levels (1,098 ± 1,831 ng/L) on the day of OR-IVM. The observation that serum AMH levels were significantly reduced on the day of IVM-OR (p = 0.005) is in accordance with previous studies showing reduced AMH levels after controlled ovarian stimulation for c-ART [21,22]. Nevertheless, although in c-ART cycles the number of small antral follicles producing AMH decreases as follicles progressively grow in response to FSH stimulation, this mechanism does not occur in IVM-cycles: in the study presented here, IVM-OR was performed when follicles had a maximal mean diameter of 10 mm. Because previous studies have shown that FSH increases estradiol levels and suppresses AMH secretion [23,24], we propose that the observed significant reduction of circulating AMH levels on the day of IVM-OR is caused by increased serum estradiol levels secondary to exogenous administration of estradiol and gonadotropins. Elevated estradiol levels also enhance hepatic SHBG synthesis and secretion, resulting in increased SHBG levels on the day of IVM-OR. TT values were also increased after mild ovarian stimulation for IVM (p = 0.002). This might be explained by the fact that the amounts of LH and hCG present in HP-hMG formulations are capable of stimulating androgen substrate production from theca cells [25].

Taking into account that decreased AMH biosynthesis following LOD restores ovulatory function through enhanced follicular sensitivity to circulating FSH levels, we speculated that a similar effect might occur following immature oocyte retrieval from small follicles. LOD has been proven to significantly reduce circulating AMH levels [26], with values remaining significantly reduced up to 6 months after the procedure [16]. The mechanic effect of LOD on polycystic ovaries may reside in destruction of small antral and pre-antral follicles, besides stroma, resulting in a relatively long term impact on circulating levels of AMH, LH, and total and free testosterone [27]. As a result of these changes, increased ovulation and pregnancy rates have been documented after LOD in patients with PCOS. Transvaginal aspiration of small antral follicles has been proposed as a method to induce ovulation in anovulatory patients with PCO [28]: in a series of 18 patients, Mio et al. demonstrated a mean ovulation rate of 52.6% in subsequent cycles after multiple antral follicular punctures. Using a similar approach, Ferraretti et al. performed transvaginal ovarian puncture in PCOS patients to improve IVF results and named this procedure “transvaginal ovarian drilling” [29]. In a case-control study in 42 patients with PCOS who had IVM treatment before conventional IVF and 48 patients with PCOS who had not had IVM before, significantly more oocytes and embryos in subsequent IVF cycles were obtained in patients who had had IVM treatment [6]. Moreover, Frantz et al. reported three spontaneous pregnancies in women with PCOS after transvaginal ovarian punctures for IVM. Although this is small case report series the authors concluded that multiple ovarian punctures for IVM in PCOS patients may have contributed to their pregnancy in the months following the IVM procedure [30]. However, although the above observations imply that multiple punctures for IVM may have an effect similar to that of LOD, no study has been conducted to provide a biological explanation for this effect. In the current study we examined the true impact of transvaginal puncture of minimally stimulated polycystic ovaries on the endocrine profile and conclude that the effect is only temporary: serum AMH concentrations had returned to levels before oocyte retrieval within two weeks after IVM-OR and the reduction of androgen levels had disappeared within three months after IVM-OR, at least in the subset of patients who had a follow-up at three months. This rather short-term effect compared to LOD might be explained by the relatively less invasive nature of ovarian puncture for the retrieval of immature oocytes, probably because of the absence of thermal energy. Hence, the new wave of developing small antral follicles that is observed as early as five days after IVM-OR [31] will result in restored steroidogenesis and AMH synthesis soon after the procedure.

In spite of its prospective design, the current study has several limitations. First, serial hormone analysis was restricted to the IVM cycle itself and only a small subset of patients were available for hormone profiling at three months’ follow-up. After one IVM cycle, the majority of patients in this study were not pregnant and proceeded with reproductive treatment shortly after this failed treatment cycle. The observation, at least in the small subset of five patients available for follow-up, that the endocrine profile was unchanged at three months’ follow-up, leads us to suggest that it is highly unlikely that the results would have been different at longer intervals. However, follow-up data were only available for five patients, which significantly limits the ability to generalize these results to a larger population.

Secondly, this study only focused on markers of the endocrine profile; the effect of ovarian puncture on ovulatory function and clinical outcomes following IVM-OR could not be investigated within the scope of this study, since the majority of patients decided to have further ART cycles shortly after the study cycle, rather than await potential effects of treatment on endocrine or ovulatory function.

Furthermore, the effect of multiple ovarian punctures for IVM on the endocrine profile described in this study has not been compared to the effect of a regular oocyte retrieval in women with PCOS undergoing a c-ART. A prospective study should be conducted in order to confirm the findings of the current study.

Our study cohort consisted of patients with PCOS, who were generally overweight or obese (mean BMI 30.3 ± 9.0 kg/m^2^) but who had relatively low serum levels of LH levels (7.9 ± 4.4 IU/L) and normal markers of hyperandrogenism (TT 0.36 ± 0.13 nmol/L, FTc 5.0 ± 3.6 pmol/L and SHBG 74.6 ± 59.1 nmol/L). In a study investigating parameters that are predictive of ovulation rates and pregnancy in 200 patients with PCOS who underwent LOD, it was shown that morbid obesity and high levels of androgens, among other parameters, are associated with low chances of response to LOD [32]. According to these findings, the patients in our study would be predicted good responders to LOD in terms of ovulatory function and clinical outcomes. Again, long-term follow-up to evaluate the effect of oocyte retrieval for IVM on ovulation rates and pregnancy could not be performed in our patients.

## Conclusions

The clinical implication from the current study is that ovarian puncture for immature oocyte retrieval appears not to have a long-term effect on the endocrine profile in patients with PCOS, contrary to what is observed after LOD. The effect of ovarian puncture appears to be transient and completely recovers three months after oocyte retrieval.

The lower efficiency of IVM systems compared to gonadotropin-driven ART precludes the widespread use of current IVM protocols. Improvement of IVM culture media could substantially enhance the potential of this technology [33], especially in patients with PCOS, in whom conventional methods of ART can be cumbersome and are associated with an increased risk of OHSS. Whether ovarian puncture for IVM treatment could enhance clinical outcomes of subsequent conventional ART treatment in patients with PCOS, remains to be confirmed. The short-term improvement of endocrine parameters related to excess follicle number and increased androgen biosynthesis in patients with PCOS might result in an increased number of oocytes and embryos in IVF cycles following IVM as observed by Agdi et al. [6]. However, the data from our study do not confirm this hypothesis.

## Competing interests

The authors declare that they have no competing interests.

## Authors’ contributions

COH, NP, EA, LG, JS and MDV contributed substantially to conception and design of the study, data acquisition, analysis and interpretation. COH, NP, EA, LG, HT, JS and MDV contributed in drafting the article or revising it critically for important intellectual content. All authors read and approved the final manuscript.
